# Alzheimer blood biomarkers: practical guidelines for study design, sample collection, processing, biobanking, measurement and result reporting

**DOI:** 10.1186/s13024-024-00711-1

**Published:** 2024-05-15

**Authors:** Xuemei Zeng, Yijun Chen, Anuradha Sehrawat, Jihui Lee, Tara K. Lafferty, Julia Kofler, Sarah B. Berman, Robert A. Sweet, Dana L. Tudorascu, William E. Klunk, Milos D. Ikonomovic, Anna Pfister, Henrik Zetterberg, Beth E. Snitz, Anne D. Cohen, Victor L. Villemagne, Tharick A. Pascoal, M. llyas Kamboh, Oscar I. Lopez, Kaj Blennow, Thomas K. Karikari

**Affiliations:** 1 Department of Psychiatry, School of Medicine, University of Pittsburgh, 3811 O’Hara Street, Pittsburgh, PA 15213 USA; 2 Department of Chemistry, University of Pittsburgh, Pittsburgh, PA 15213 USA; 3 Department of Pathology, School of Medicine, University of Pittsburgh, Pittsburgh, PA 15213 USA; 4 Department of Neurology, School of Medicine, University of Pittsburgh, Pittsburgh, PA 15213 USA; 5 Geriatric Research Education and Clinical Center, VA Pittsburgh HS, Pittsburgh, PA USA; 6 Department of Psychiatry and Neurochemistry, Institute of Neuroscience and Physiology, The Sahlgrenska Academy, University of Gothenburg, Mölndal, Sweden; 7 Clinical Neurochemistry Laboratory, Sahlgrenska University Hospital, Mölndal, Sweden; 8 Department of Neurodegenerative Disease, UCL Queen Square Institute of Neurology, London, UK; 9 UK Dementia Research Institute at UCL, London, UK; 10 Hong Kong Center for Neurodegenerative Diseases, Hong Kong, China; 11 Department of Medicine, School of Medicine and Public Health, University of Wisconsin-Madison, Madison, WI USA; 12 Department of Human Genetics, School of Public Health, University of Pittsburgh, Pittsburgh, PA 15213 USA

**Keywords:** Blood biomarker, Standardization, Preanalytical factors, Alzheimer’s disease, Biobanking, Neurodegeneration

## Abstract

Alzheimer’s disease (AD), the most common form of dementia, remains challenging to understand and treat despite decades of research and clinical investigation. This might be partly due to a lack of widely available and cost-effective modalities for diagnosis and prognosis. Recently, the blood-based AD biomarker field has seen significant progress driven by technological advances, mainly improved analytical sensitivity and precision of the assays and measurement platforms. Several blood-based biomarkers have shown high potential for accurately detecting AD pathophysiology. As a result, there has been considerable interest in applying these biomarkers for diagnosis and prognosis, as surrogate metrics to investigate the impact of various covariates on AD pathophysiology and to accelerate AD therapeutic trials and monitor treatment effects. However, the lack of standardization of how blood samples and collected, processed, stored analyzed and reported can affect the reproducibility of these biomarker measurements, potentially hindering progress toward their widespread use in clinical and research settings. To help address these issues, we provide fundamental guidelines developed according to recent research findings on the impact of sample handling on blood biomarker measurements. These guidelines cover important considerations including study design, blood collection, blood processing, biobanking, biomarker measurement, and result reporting. Furthermore, the proposed guidelines include best practices for appropriate blood handling procedures for genetic and ribonucleic acid analyses. While we focus on the key blood-based AD biomarkers for the AT(N) criteria (e.g., amyloid-beta [Aβ]40, Aβ42, Aβ42/40 ratio, total-tau, phosphorylated-tau, neurofilament light chain, brain-derived tau and glial fibrillary acidic protein), we anticipate that these guidelines will generally be applicable to other types of blood biomarkers. We also anticipate that these guidelines will assist investigators in planning and executing biomarker research, enabling harmonization of sample handling to improve comparability across studies.

**Supplementary Information:**

The online version contains supplementary material available at 10.1186/s13024-024-00711-1.

## Background

Alzheimer’s disease (AD), the most common form of dementia, poses significant economic and social burden on affected individuals, as well as their families, caregivers, communities, and healthcare systems worldwide [1]. An estimated excess of 50 million people are living with AD globally, including 6.7 million in the United States. By 2050, these numbers are expected to rise to 152 million and 13.8 million, respectively [2, 3]. Neuropathologically, AD is characterized by two hallmark lesions in the brain; amyloid-β (Aβ) plaques and tau neurofibrillary tangles [4, 5]. Most individuals with brain pathophysiological evidence of AD clinically show progressive cognitive impairment [6]. Unfortunately, despite decades of research and numerous clinical trials, AD remains difficult to treat, with only a few FDA-approved drugs available for treatment [1]. Among them, lecanemab, donanemab and aducanumab, humanized antibodies designed to reduce the amyloid plaque burden, are the only ones expected to provide disease-modifying therapy [7–9]. The other drugs are palliative treatments that reduce the symptoms temporarily but are not directed toward preventing or slowing disease progression.

The slow pace of AD drug development is partly due to a lack of accessible and cost-effective biomarkers for participant enrollment and stratification in clinical trials. The National Institute on Aging and the Alzheimer’s Association (NIA-AA) research framework recommends the use of biomarker criteria for amyloid pathology, tau pathology, and neurodegeneration [AT(N)] for a biological definition of AD [10]. However, these assessments are currently performed using expensive, time-consuming, and sometimes invasive procedures with limited global accessibility such as magnetic resonance imaging (MRI), positron emission tomography (PET) scans, and/or cerebrospinal fluid (CSF) biomarkers [11–13], which are unsuitable for large-scale clinical applications and population screenings. It has been estimated that screening a single participant for AD clinical trials with PET and MRI could cost at least US $8,000 [14]. Given the typically high screen-failure rate (percentage of screened participants not meeting the enrollment criteria), it is not surprising that participant screening may cost 50–70% of total per-participant costs [14]. These costs could be prohibitive for large-scale clinical trials. In terms of clinical applications, imaging all patients with suspected cognitive impairment due to AD using PET and MRI would be difficult to achieve due to the low throughput and the limited availability of the specialized facilities and expertise needed to administer and interpret these tests [13].

To address this issue, there is a growing need to develop less invasive, more cost-effective, and scalable biomarkers that can reliably identify AD pathology. Blood-based AD biomarkers are a desirable choice due to availability of blood specimens through routine clinical practice and research programs. In clinical trials, blood biomarkers have already shown utility as pre-screening measures to streamline the identification and inclusion of individuals who fit pre-defined criteria for biological evidence of disease [15, 16]. Importantly, clinical prescriptions of the recent FDA-approved anti-amyloid drugs require prior confirmation of brain amyloidosis. However, since amyloid PET and CSF Aβ42/Aβ 40 assessments are not feasible in many hospital settings, blood biomarkers would be very useful proxies.

The development of such biomarkers has been hindered by the extreme complexity of the blood proteome, low biomarker abundance, and signal dilution from peripheral tissues. However, significant advances have been achieved in the past decade, benefiting partly from the development of ultra-sensitive immunoassays and high-performance mass spectrometry technology platforms [17–20]. Blood-based biomarkers with high potential of providing accurate assessment of the AT(N) criteria include the Aβ42/40 ratio for amyloid pathology, phosphorylated tau (p-tau) for tau pathology, and neurofilament light-chain (NfL) and brain-derived tau for neurodegeneration/axonal injury [11, 21]. In addition, plasma glial fibrillary acidic protein (GFAP), an indicator of reactive astrogliosis often associated with brain Aβ plaques, has also been proposed as an early marker for amyloid pathology [22–25]. 

The anticipated next stage in the development of highly sensitive and specific blood biomarkers for AD is to employ them in real-world settings for clinical diagnosis, population studies, and eligibility screening for therapeutic trials. However, a major challenge facing the field is the need for increased standardization of collection, processing, and storage procedures. Another important challenge is the need for agreed-upon procedures to monitor and maintain long-term stability in the biomarker measurements, especially since none of the blood-based AD biomarkers in use currently has certified reference measurement procedures. These obstacles must be overcome before blood-based biomarkers can be effectively adopted in clinical and research-based settings, and these measurements can be appropriately harmonized. A survey of studies across fifteen centers revealed variations in sample processing, such as the time of day for collection, fasting status, time from collection to centrifugation, the temperature at various steps, and centrifugation parameters [26]. The lack of standardization can introduce measurement variations, reducing clinical reliability and making it challenging to compare results across laboratories and establish clinical thresholds. These variations can be introduced at three phases: preanalytical, analytical, and post-analytical. The advancement of analytical technologies, such as automation in sample preparation, has dramatically reduced errors in the analytical phase. It is now thought that the preanalytical stage is the most error-prone phase (over 60%), followed by the post-analytical phase (over 20%) [27].

Decades of research have led to the development of a consensus protocol for standardized collection and biobanking of CSF-based AD biomarkers [28], which has been key to the much improved inter-laboratory reproducibility of the core CSF biomarkers in recent years [29]. Blood-based AD biomarkers often have smaller effect sizes than the corresponding CSF biomarkers (with the exception of GFAP) [25], possibly due to signal attenuation caused by counterpart proteins secreted or biomarkers sequestered by peripheral tissues and the increased biological complexity of blood [13, 30] (further discussed below). Therefore, minimizing preanalytical variations is even more critical for blood-based AD biomarkers. O’Bryant et al., in 2015, proposed a set of guidelines to standardize blood sample collection [31]. Yet, the evidence evaluating defined preanalytical factors was limited at that time. Since then, many research studies have been published, and an updated evidence-based plasma handling standardized operating procedure (SOP) was proposed in 2022 by Verberk et al. [26]. However, this SOP was limited to preanalytical factors and was directed at experienced blood biomarker laboratories and scientists. As blood biomarkers become more widely available and simplified commercial technologies get increasingly accessible, fundamental guidelines that: (1) take into account the preanalytical, analytical, and post-analytical pipeline; (2) accommodate the needs of investigators new to the blood biomarker space; and (3) are applicable to both traditional (blood collection and assessments in a clinical setting) and non-traditional (home collections, population-based evaluations, resource-limited settings) environments, are needed.

**Fig. 1:**
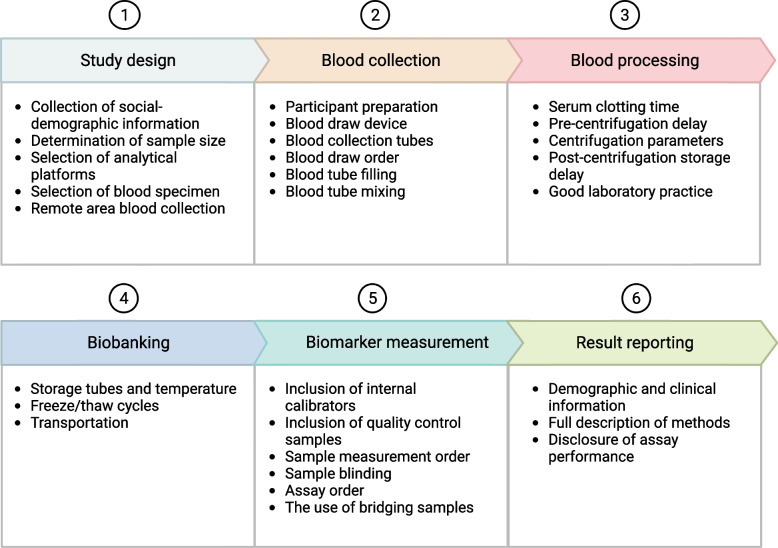
Proposed general workflow for conducting high-quality blood AD biomarker research and the important considerations associated with each step. The major steps are divided into six, namely study design, blood collection, blood processing, biobanking, biomarker measurement and results reporting. The important considerations under each step are listed in the Figure. Detailed discussions are provided in the text

Standardization of blood AD biomarker research across sites, studies and investigators must consider several steps, including study design, blood collection, blood processing, biobanking, biomarker measurement, and result reporting (Fig. 1). This review aims to expand on earlier guidelines by employing the evidence base from recent research findings and our own experiences to provide a detailed description of the general considerations associated with each of these steps. Furthermore, we present an easy-to-follow SOP to aid in the design and implementation of high-quality blood-based AD biomarker research projects, covering the preanalytical, analytical, and post-analytical phases. It is worth noting that although we focus on the key blood-based AD biomarkers for the AT(N) criteria (e.g., Aβ40, Aβ42, Aβ42/40 ratio, total-tau (t-tau), p-tau, NfL, brain-derived tau [BD-tau] and GFAP), we anticipate that these guidelines will generally be applicable to other types of blood biomarkers as well as discovery proteomic investigations.

## Considerations during study design

### Item 1: collection of sociodemographic, lifestyle, and health information

Numerous sociodemographic, lifestyle, health, and environmental factors have been associated with an increased risk of AD [32–34]. For example, age, education, sex, race, creatinine levels, concomitant medication (including anti-amyloid therapies), medical history, and comorbidities such as diabetes, hypertension, impaired kidney function, liver diseases, and cardiovascular diseases have been found to be significantly associated with blood AD biomarkers levels in some research cohorts compared with unaffected controls [7, 35–43]. Additionally, conditions such as pregnancy, menopausal symptoms and hormone therapy use are critical as biomarker changes have been reported [44, 45] but require further investigation. While it may not be feasible to include all demographic and clinical information, we recommend recording as much information as possible during subject recruitment and through the medical record extraction to prevent over-/under-interpretation of results and to enable adjustment of reference ranges according to population characteristics if needed. Importantly, the collection, processing, storage, and application of personal data must conform with the prevailing ethical guidelines and legal frameworks in the countries concerned. For example, there is the General Data Protection Regulation (GDPR) in Europe and Health Insurance Portability and Accountability Act of 1996 (HIPAA) in the United States.

### Item 2: sample size consideration

Sample size planning is critical in studies evaluating the diagnostic and prognostic capabilities of blood AD biomarkers and the impact of covariates. Insufficient sample size reduces the power to detect true significance. In contrast, excess sample size may magnify the importance of clinically non-meaningful differences, increase costs, prolong the study, and potentially expose more participants to needless evaluations [46]. Blood AD biomarkers often have smaller effect sizes than CSF biomarkers due to the expected dilution effect from peripheral tissues. Aβ42/40 ratio, for example, has reduced difference between Aβ+ *vs.* Aβ- individuals from ~ 40% in CSF to ~ 10% in blood [13, 25, 30]. Similarly, p-tau fold change is comparatively decreased from 166% in CSF to 85.6% in the blood [13], despite plasma p-tau having larger effect sizes than plasma Aβ42/40 ratio when compared directly [25, 47]. Therefore, blood AD biomarker studies will require larger sample sizes than CSF biomarker studies. To determine the optimal sample size, one must consider factors such as expected effect size, population variance, desirable type I and type II error rates, participant dropout rate for longitudinal studies, and adjustment of covariates [46]. It is important to note that sample size calculation can become challenging for complex studies and will require statisticians’ advice during study design.

### Item 3: selection of analytical platforms

Blood AD biomarkers tend to be near or below the detection limit of traditional enzyme-linked immunosorbent assays (ELISAs). However, in the past decade, many technologies have emerged that have significantly accelerated research in this area [18, 19, 48–62]. Among them, Single molecule array (Simoa), mass spectrometry, as well as immunoassay technologies available on platforms such as the Elecsys and Cobas systems from Roche Diagnostics, Meso Scale Discovery (MSD), and immunomagnetic reduction (MagQu) are the most used. Recently, the Lumipulse G system, widely used for running electrochemiluminescence (ECL) immunoassays in CSF samples for research and in vitro diagnostic purposes, has also moved into the blood biomarker space, with assays now available for plasma Aβ peptides, NfL, p-tau181 and p-tau217 [63]. In addition, Ella, a novel platform for running multi-analyte automated microfluidic immunoassays (Simple Plex™) has now entered the AD biomarker field, with assays available for plasma NfL [64, 65]. Emerging platforms include Nano Mosaic and NULISA. We have summarized in Table 1 the commonly used platforms for the key AD blood biomarkers, but the list is not meant to be exhaustive. For more detailed descriptions, readers should refer to recent review articles [20, 66–68] and references therein.

**Table 1:** Frequently used analytical platforms for core AD blood biomarkers and their analytical characteristics

Platform	Technology	Core AD biomarker (Assay range in pg/ml)	Remarks
IP-MS-Shim [18]	IP-MALDI-TOF	Aβ40 (40–640)^a^, Aβ42 (11–180)^a^	• Sample volume: 250 μL • Also measure other Aβ peptides including Aβ38, Aβ39, Aβ3-40, and APP669-711.
IP-MS-WashU [57, 69]	IP-LC/MS/MS	Aβ40 (11–26,400)^b^, Aβ42 (2–3,920)^b^, various p-tau and np-tau variants	• Two clinically approved assays were developed based on this platform for identifying brain amyloid plaques in cognitively impaired patients aged 55 and above: PrecivityAD™ measures Aβ42/40 and ApoE proteotype, while PrecivityAD2™ also includes the p-tau217/np-p-tau217 ratio. • Sample volume: 450 μL for Aβ peptides and 1,000 μL for tau variants.
LC-MS-Arc [70]	LC-MS/MS	Aβ40 (50–1,000)^a^, Aβ42 (10–200)^a^	• Antibody-free LC-MS • Sample volume: 200 μL
IP-MS-UGOT [54, 71]	IP-LC/MS/MS	Aβ40 (50–800)^a^, Aβ42 (10–2000)^a^, various p-tau and np-tau species	• Sample volume: 250 μL for Aβ peptides and 1,000 μL for tau
Quanterix (HD-X)	Bead-based Simoa	Aβ40 (4.08–280), Aβ42 (1.51–100), p-tau181 (8–1280), p-tau217 (0.007–30), t-tau (0.248–360), NfL (1.38–2000), GFAP (0.248–360)	• Up to 4 plex. • Sample volume: 25 to 38 μL per analysis plus 30 μL dead volume
Lumipulse G1200	ECL	Aβ40, Aβ42, p-tau181, p-tau217, NfL	• Singleplex • Sample volumeL 70 to 130 μL per analysis plus 100 μL dead vlolume
Mesoscale Discovery	ECL	p-tau181 (0.46–990), p-tau217 (5.90–2,400), p-tau231 (15.0–15,000), t-tau (0.12–160), NfL (5.40–3,600), GFAP (0.32–850)	• Singleplex for p-tau; 3-plex for NfL, GFAP and t-tau • V-PLEX Aβ Peptide Panels exhibit sub-pg/ml sensitivity, but they haven’t been validated in human plasma. • Sample volume: 25 μL per analysis.
Roche Elecsys (Cobas)	ECL	Aβ40, Aβ42, NfL, p-tau181, p-tau217, GFAP	• Comercial assays are validated only in CSF samples, with the exception of NfL. Prototype assays are available for other biomarkers [72].
Ella [65]	Microfluidic immunoassay	Aβ40 (24.5–2,400), Aβ42 (6.55–1,600), GFAP (72.1–110,000), NfL (2.7–10,290)	• Can simutenously measure up to 8 plexes • Sample volume: 25 μL per analysis
Alamar (ARGO HT) [73]	NULISA™	Aβ40, Aβ42, p-tau181, p-tau217, p-tau231, NfL, GFAP, t-tau	• Both singleplex or multiplex assays are available. • NULISAseq CNS Panel targets ~ 120 biomarkers whcih includes all listed biomarkers and other key proteins associated with neurodegenerative disorders.Use 10/20 μL per analysis. • Exhibits fg/ml sensitivity with large dynamic range (up to 12 logs).
MagQu [74]	IMR	t-tau (1–100), Aβ40 (1- 200), Aβ42 (1–100), p-tau181 (0.02–200), NfL (0.01 to 100), GFAP (1 to 100)	• Singleplex • Sample volume: 40 to 60 μL per analysis. • In contrast to other immunoplatform assays, MagQu employs only a single antibody as a probe for each biomarker, rather than a pair of capture and detection antibodies. • MagQu assays for Aβ peptides often exhibited different trend comapred to other immunoassay platforms [75].

*ECL* Electrochemiluminescence, *Simoa* Single Molecule Array, *IP* Immunoprecipitation, *IMR* Immunomagnetic Reduction, *NULISA* NUcleic acid Linked Immuno-Sandwich Assay, *np-tau* Non-phosphorylated tau, *np-p-tau217* Non-phosphorylated tau corresponding to p-tau217 region

The assay ranges were obtained from product inserts available on the vendors’ websites, except for: ^a^sourced from the review article by Korecka M. and Shaw LM [76]; ^b^obtained from prior publication by Keshavan A et al. [54]

Extensive studies have been conducted to examine associations of blood biomarkers with AD pathology and compare the performance of different platforms. While a comprehensive review of the literature is beyond the scope of this work, a brief summary of the key findings is provided below:

- Amyloid plaques, one of the primary pathological features of AD, consist mainly of amyloid beta peptides [77, 78]. While CSF Aβ42/40 has been used in clinical settings to assess brain Aβ plaques, the association of blood Aβ42/40 with AD pathologies has been controversial [79, 80]. Several immunoassays and MS assays are available to measure blood Aβ peptides [20], but overall, there is low inter-platform reproducibility [75, 81]. MS assays generally exhibit superior predictive power for brain Aβ compared to immunoassays, possibly due to higher specificity obtained through MS assays [81].
- CSF t-tau is a biomarker for neurodegeneration or neuronal injury [82]. However, plasma t-tau shows low correlation with CSF t-tau due to potential contamination with tau from peripheral sources [83, 84]. Improved plasma t-tau assays have been reported recently [85, 86]. In addition, recently developed Simoa assay targeting brain-derived tau showed a better correlation with CSF t-tau and improved biomarker performance [21].
- CSF p-tau is a biomarker for neurofibrillary tangles [15, 87, 88]. Despite their low abundance in the blood, several assays are available to measure p-tau species in the blood [13, 89–92]. Unlike plasma Aβ assays, p-tau assays exhibited overall strong inter-platform concordance [20, 93–96]. P-tau181, p-tau217, and p-tau231 are the most widely studied p-tau species. P-tau212 is a new marker recently reported [97]. Different p-tau species might increase at different stages of the AD continuum [91, 98]. Unlike their CSF counterparts, blood p-tau exhibits better association with Aβ plaques rather than neurofibrillary tangles.
- GFAP is a biomarker for reactive astrogliosis [99], a cellular response often associated with brain Aβ plaque pathology in AD [100]. Plasma GFAP positively correlated with Aβ burden and tau pathology in AD [101, 102]. Plasma GFAP level may be impacted by non-AD brain injuries and is an FDA-approved biomarker for detecting intracranial lesions after brain injury [103].
- Neuronal damage/injury leads to elevated secretion of NfL into the extracellular space [104]. Although non-AD specific, NfL is an excellent biomarker for neurodegeneration to monitor the disease progression of AD patients [105, 106]. Head-to-head comparison of Simoa and Ella assays in a multiple sclerosis cohort demonstrated a strong correlation between the platforms [64, 65]. Plasma/serum brain-derived tau showed stronger specificity to AD pathophysiology versus related non-AD disorders. 

### Item 4: selection of blood specimen

Both plasma and serum have been utilized for measuring AD biomarkers. Studies comparing AD biomarker levels in serum and plasma have shown that some analytes, including Aβ peptides, t-tau, BD-tau and multiple p-tau species, are present at lower levels in serum, possibly due to a loss from clot trapping [26, 107–111]. This makes it more challenging to measure such biomarkers in serum, especially for individuals whose biomarker levels are close to the lower detection limit. Nonetheless, biomarkers such as p-tau231, p-tau181 and BD-tau have been shown to have equivalent diagnostic accuracies in plasma and serum [21, 89, 107, 109, 111]. P-tau217 [112] and p-tau212 [97] are currently measurable in plasma but not serum. It is important to note that the choice of the blood specimen depends on the overall research objectives and sample availability. For example, serum may be a better choice for studies that evaluate the integrity of the blood-brain barrier since the CSF/serum albumin ratio is a well-established indicator of blood-CSF barrier function [113]. Additionally, serum is more widely used in hospital systems, with more clinical tests using serum instead of plasma as the specimen, according to the Mayo Clinic 2023 Test Catalog [114].

On the other hand, many research cohorts collect plasma instead of serum. Ethylenediaminetetraacetic acid (EDTA), heparin, and citrate are the most commonly used anticoagulants in clinics for plasma collection [115]. EDTA is the most universal in AD biomarker research [26]. Several studies have suggested that citrate plasma has lower levels of several biomarkers, including Aβ peptides, NfL, GFAP, and t-tau, compared with EDTA and heparin plasma [116, 117]. However, studies comparing heparin vs. EDTA have generated mixed results. For example, one study found most biomarkers to be more abundant in heparin plasma than EDTA plasma [107], while another found higher levels of t-tau and p-tau181 but similar levels of Aβ40 and Aβ42 in heparin compared with EDTA plasma samples [118]. Rózga et al., on the other hand, reported that the levels of t-tau were significantly lower in heparin plasma [116].

Regardless of the type of blood specimen chosen, it is important to use the same type of specimen throughout the study. Although biomarkers may show a similar trend in different specimens, they are not necessarily linearly correlated in samples from all individuals. For example, despite strong correlations and similar diagnostic accuracy between paired serum and plasma p-tau levels, Kac et al. observed larger disagreements in samples with lower p-tau concentrations [109].

### Item 5: blood collection from remote areas, under-resourced settings, or home care

Advanced laboratory equipment, such as ultra-low temperature freezers and centrifuges, are typically required for the processing of traditional venipuncture-based blood specimen. This can create significant obstacles for community-based studies utilizing home sampling, as well as for studies in remote areas, where access to such equipment may be limited. In addition, venipuncture may be difficult and painful for individuals with small or fragile veins [119]. To overcome these challenges, some research initiatives have explored alternative blood collection methods. For instance, Walter et al. compared Aβ40 and Aβ42 levels in conventional venous blood *vs.* capillary blood collected by finger insertion using microvettes. They found a good correlation between the two specimen types, despite slightly lower levels in capillary blood [120]. Similarly, Lombardi et al. and Simrén et al. investigated the use of dried blood spots (DBS) and dried plasma spots (DPS) for NfL measurement and found that NfL levels in both DBS and DPS samples correlated strongly with those in EDTA plasma samples with a stronger correlation observed for DPS samples [121, 122].

## Considerations during blood collection

### Item 1: preparation of participants for the blood draw

Studies examining the impact of pre-blood draw activities of participants, such as fasting, physical exercise, medication use, and the time of day for blood collection, are limited. However, preliminary evidence shows that some of these factors can impact blood biomarker levels. For example, Rózga et al. found that the levels of Aβ40 and Aβ42 were 5–9% higher in blood samples collected in the afternoon compared with those collected in the morning, with the opposite trend noted for t-tau [116]. Meyer et al. found significantly higher Aβ40 and Aβ42 levels in non-fasting blood when measured with Simoa assays [123]. Signal variation from plasma samples obtained on consecutive days from the same individual or within a cohort, which allows for the evaluation of short term fluctuation, have also been reported [124].

To minimize potential bias arising from these effects, we recommend pre-defining the participant preparation protocol upfront and following it throughout the study. Ideally, blood should be drawn at the same time of the day for all participants with the same fasting status throughout the study. If practical in the population under study, fasting blood samples may be more reliable in general. It is also important to record information such as blood draw time, fasting status, date and time of last meal, hours of sleep the previous night, pre-blood draw exercise activity, and medication use, to facilitate downstream interpretation of results.

### Item 2: blood draw devices

Blood can be collected from participants in various ways, including venipuncture using an evacuated system (Vacutainer®), a syringe, butterfly needles, intravenous (IV) catheters, fingersticks, or heelsticks. The choice of blood draw methods depends on factors such as patient characteristics, the type of tests to be performed, and the preference and experience of clinical staff. Venipuncture using a needle and vacutainer tubes are the most used for routine blood draw. It is important to note that the devices used for the blood draw may influence the blood sample quality. For example, IV catheters and smaller-bore needles are sometimes used for patients with hard-to-access veins or when multiple blood draws over an extended period are needed. However, both have been found to have a higher hemolysis rate [125, 126]. Additionally, lubricant coating and needle material, if released into the blood, can potentially contaminate the specimens, which have been shown to affect antigen-antibody binding in some immunoassays [127]. There is a lack of published research on the impact of blood draw devices on blood AD biomarker levels, aside from the type of blood collection tubes used (described below). To harmonize the blood collection procedures across different labs, O’Bryant et al. recommended using 21-gauge needles for blood draw in adults [31]. When possible, new straight needle venipuncture is preferred over the IV start. Any variation from standard procedures should be carefully documented.

### Item 3: blood collection tubes

**Fig. 2:**
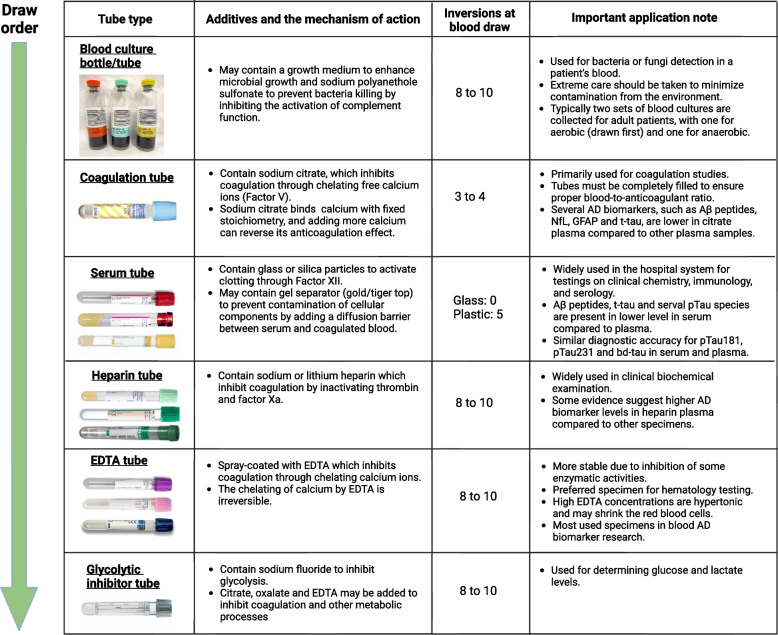
Commonly used human blood collection tubes, their draw order, additives, and important application notes. The numbers of inversions are based on BD’s recommendations for Vacutainer® tubes

Most biomarker studies use evacuated tube systems for blood collection. There are many different brands of evacuated tube systems available, including BD’s Vacutainer®, Sarstedt’s Monovette®, and Greiner Bio-One’s VACUETTE®. Different blood collection tubes vary in materials, shape, size, additives used, and safety features. The color of the tube closure typically indicates the additives. We have listed some commonly used blood collection tubes and their intended clinical applications in Fig. 2. Various components of the evacuated tubes, such as their surface coating, stopper materials, stopper lubricants, gel separators, and additives, can interfere with clinical laboratory assays and are potential sources for preanalytical variation [115, 127, 128]. For instance, gel separators – inert gels used as barriers for better separation of serum or plasma from cells/clots after centrifugation – have been shown to absorb blood constituents and interfere with various clinical tests for therapeutic drug monitoring [129]. It is worth noting that several studies indicated that AD biomarkers, especially Aβ peptides in the CSF matrix, may exhibit varying absorption rates to tube walls based on the materials they are made of. Specifically, there is a higher overall loss when polystyrene tubes are utilized [130–132]. Pre-treating the tubes with detergent Tween-20 might mitigate the absorption [133, 134]. However, currently, there is no evidence to suggest that the primary materials used in blood tubes have any effect on AD biomarker levels [116, 135]. To reduce the risk of variability caused by using different types of tubes, we suggest using the same brand of tubes consistently throughout the studies and limiting the number of lots to as few as possible.

### Item 4: blood draw order

Different blood collection tubes contain distinctive additives. To minimize the impact of additive cross-contamination when collecting samples from a single blood draw into different types of tubes, the Clinical and Laboratory Standards Institute [136] CLSI; https://clsi.org/) recommended a specific blood draw order (as shown in Fig. 2): (1) blood culture tube or bottle; (2) sodium citrate tube; (3) serum tube; (4) heparin tube; (5) EDTA tube; (6) sodium fluoride/potassium oxalate glycolytic inhibitor tube. Following the recommended blood draw order is crucial to avoid carry-over additives that may result in inaccurate results. For example, if an EDTA tube is drawn before a serum tube, some of the EDTA may carry over into the serum tube and interfere with the coagulation.

### Item 5: blood collection tube filling height

Some collection tubes, such as those for plasma collection, contain spray-coated or liquid additives. Therefore, different tube filling heights may cause variations in the blood-to-additive ratio, potentially influencing the protein composition. In support of this, Rózga et al. found a lower level of plasma t-tau when K_2_-EDTA tubes were filled only to 50% compared to 100% [116]. Therefore, to minimize variation, we recommend adhering to manufacturers’ recommendations for blood volume filling to maintain a consistent additive-to-blood ratio among all samples.

### Item 6: proper mixing of blood samples

For blood collection tubes containing additives, it is crucial to gently invert the tubes immediately after the blood draw to ensure proper mixing of the additive with the blood. Failure to do so may result in non-homogenous samples and the formation of microclots or residual fibrins that can obstruct the sample probe of analytical instruments. The number of required inversions varies by tube type. We suggest following the manufacturer’s guidelines for the mixing. The recommended number of inversions for BD’s Vacutainer® tubes is listed in Fig. 2.

## Considerations during blood processing

Both serum and plasma are liquid components derived from blood after separating the blood cells, typically through centrifugation. The main difference is that serum is collected from clotted blood, while plasma is collected without clotting through anti-coagulants, thus retaining the clotting factors. Apart from the variations mentioned above for blood collection, several factors during blood processing, including pre-centrifugation delay time, centrifugation conditions, post-centrifugation storage delay, and temperature at various processing steps, may also contribute to the pre-analytical variation of the resulting blood specimens. In the following sections, we summarize current research findings on the impact of these variables and provide general guidelines for blood processing. Additionally, we have included a detailed step-by-step SOP for collecting plasma from EDTA tubes in Additional file 1.

### Item 1: serum clotting time

Harmonizing SOPs for serum collection can be challenging, partly because of the difficulty in setting the optimal clotting time. Insufficient clotting may lead to the formation of residual fibrin, which may clog the biomarker-measuring instruments [137]. In contrast, prolonged clotting may lead to cell lysis, resulting in serum contamination with cellular components [138]. The ideal clotting time varies not only by tube type but also by patient characteristics. Plain red top tubes (glass with no additive or plastic coated with silica as clot activator) and serum separator tubes (SST; gold top or tiger top) with clot activators and gel separators are commonly used in the clinics for serum collection. BD Diagnostic recommends a 30-min clotting time for SST and a 60-min clotting time for the red top tubes. Patients with certain diseases, such as liver diseases and multiple myeloma, or those on anticoagulant therapy, may require a longer clotting time. It has therefore been recommended that blood samples should be left to sit upright at room temperature for at least 30 min but no more than 60 min to allow clots to form and minimize the interference of blood cell lysis [31].

### Item 2: pre-centrifugation delay time

Several studies have investigated the impact of pre-centrifugation delay on AD blood biomarker measurements. A long delay has been associated with a more significant decrease in biomarker levels. This impact can be mitigated by storing blood at 4 °C rather than room temperature prior to centrifugation [26, 116, 120]. Among the AD blood biomarkers, Aβ peptides are particularly susceptible to loss from pre-centrifugation delay. Their levels drop in a time-dependent manner when stored at room temperature [139]. To minimize interference of blood cell lysis and protein degradation, it is recommended completing the whole process within 2 h [31] and, if not feasible, keeping the blood refrigerated for no more than 24 h [26]. However, the shorter the pre-centrifugation delay, the better for all the blood biomarkers.

### Item 3: centrifugation settings, including speed, time, and temperature

Optimal centrifugation settings are crucial for obtaining high-quality serum/plasma samples. Prolonged or excessive-speed centrifugation may cause blood cell lysis, while centrifugation that is too short and/or at an insufficient speed may result in incomplete separation of serum/plasma from blood cells [140]. Centrifugation settings may vary by blood collection tube type. For example, coagulated tubes require longer centrifugation than plasma tubes to ensure complete serum separation from the clot (CLSI H21). According to a recent survey [26] the common practice in the blood AD biomarker field is to centrifuge for 5–15 min at 1500–3000 xg. However, there are still very limited studies evaluating the impact of centrifugation parameters on AD blood biomarker measurements. A preliminary investigation found no significant difference between room temperature and 4 °C centrifugation for most AD biomarkers except t-tau, whose abundance was lower with 4 °C centrifugation [26].

### Item 4: post-centrifugation storage delay

Post-centrifugation delay may also contribute to a decrease in biomarker abundance, although the rate of decline appears slower than during pre-centrifugation delay [26, 120]. Similarly, keeping samples on wet ice while waiting for storage has been found to greatly mitigate the impact of storage delay.

### Item 5: good laboratory practice (GLP)

Adhering to GLP during blood processing is crucial for ensuring the safety, quality, and integrity of research studies. Below are some key practices that should be followed:

- All blood samples and associated collection devices should be considered potentially infectious, and proper personal protective equipment (PPE) should always be used to minimize exposure risk.
- To protect the confidentiality of research participants, personal information should not be included on specimen labels. To avoid sample mix-up, all tubes should be clearly labeled, preferably using printed labels or barcodes rather than handwritten ones. This labeling should be done in advance of the participants’ visits for blood collection.
- Good pipetting skills are essential for ensuring high sample quality. When pipetting plasma/serum from the blood collection tubes, gently draw the liquid from the top and gradually move the pipette down with the liquid. It is important to avoid disturbing the buffy coat and the cell layers in the plasma tubes and clots in the serum tubes. If allowed, leave the bottom ~ 10% of plasma/serum behind to prevent cross-layer contamination.
- If plasma/serum samples are to be aliquoted into more than one tube, it is important to transfer them from the blood collection tubes to a second, intermediary tube (such as low protein binding conical tubes) after centrifugation. Before aliquoting, the samples should be mixed by inverting the conical intermediary tube or pipetting up and down multiple times to ensure homogeneity. Direct aliquoting from the blood collection tubes right after centrifugation may lead to heterogeneity among aliquots due to the impact of centrifugation forces.
- Hemolysis significantly deteriorates sample quality and is the primary cause of unusable specimens for clinical assays [140]. Therefore, samples should be inspected for signs of hemolysis which may impact the assay results. We recommend using a quick reference chart (such as the CDC Hemolysis Reference Palette) to record the hemolysis scale during specimen collection and checking the influence of hemolysis during data analysis.

### Item 6: general procedures for serum collection

**Fig. 3:**
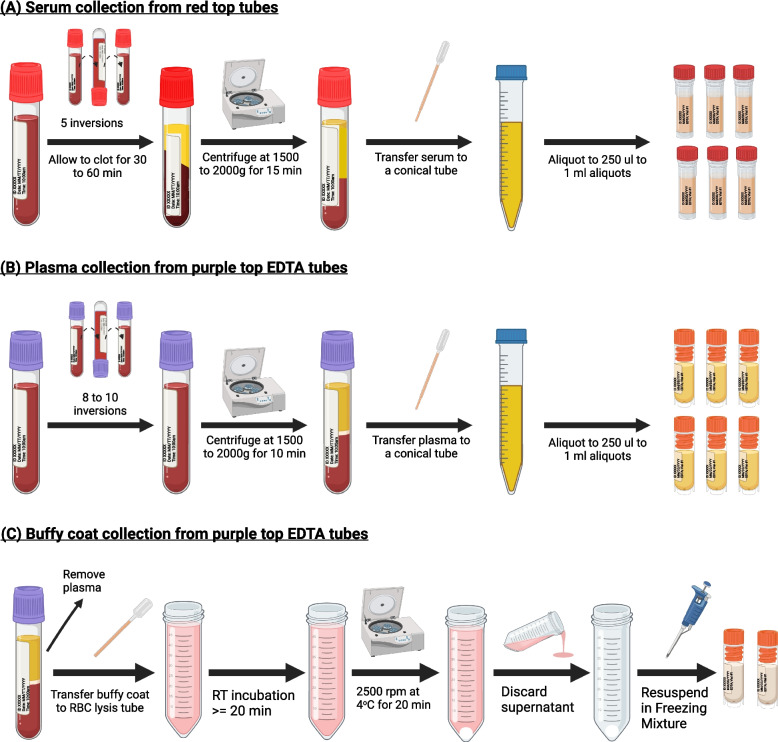
General procedures for serum (**A**), plasma (**B**), and buffy coat (**C**) collection from whole blood

Figure 3A illustrates the general procedures for collecting serum. We recommend collecting at least 5 ml of blood, yielding approximately 2.5 ml of serum to ensure sufficient specimens for multianalyte measurement. To minimize the impact of freeze/thaw, samples should be aliquoted. Both plain red-top tubes and serum separator tubes are commonly used for serum collection. Other than glass plain red top tubes, all other tube types require five inversions immediately after the blood draw to mix the blood with clot activators. Below are the general procedures for serum collection.

- **CRITICAL: If not using the glass red top tubes, phlebotomists should gently invert the blood tubes 5 times immediately after blood draw.**
- **CRITICAL: Place the filled blood collection tubes upright at room temperature for 30 to 60 min to allow the clot to form.**
- **CRITICAL: If the blood is not centrifuged immediately after the clotting time (30 to 60 min at room temperature), the tubes should be refrigerated (4 °C) for no longer than 2 h.**
- Centrifuge clotted tubes balanced by weight for 10 min at 1500 to 2000 × g at 4 °C.
- Use the disposable transfer pipette to transfer the serum (top layer) to a 15 mL conical tube (or 50 mL conical tube if collecting 30 to 100 mL of blood). Be careful not to disturb the clot containing red blood cells, white blood cells, platelets, etc.
- If more than one tube is collected, combine the serum samples from all tubes into the same conical tube.
- Gently invert the conical tube 8–10 times to mix. Aliquot 250 μl to 1 ml into labeled microtubes or cryovials with O-ring-sealed screw leads. Residual aliquots can be saved and pooled as QC samples for repeated analysis.
- Store all aliquots upright in a specimen box in an -80 °C or colder freezer.

### Item 7: general procedures for plasma collection

Figure 3B depicts the general procedures for collecting plasma. We recommend using EDTA or heparin tubes for blood collection, filling the tubes until the vacuum is exhausted, and following the procedures below.

- **CRITICAL: Immediately after blood collection, gently invert/mix (180-degree turns) the EDTA tubes 8–10 times. Place the tubes upright on a rack until centrifugation.**
- **CRITICAL: It is advisable to store collected blood at 4 °C instead of at room temperature before centrifugation. Blood samples should be centrifuged within 2 h of blood collection to minimize degradation of AD biomarkers.**
- **CRITICAL: In case of unavoidable prolonged centrifugation delay, place blood samples in the refrigerator for no more than 24 h. Avoid direct contact of blood tubes with ice to minimize cell lysis.**
- Centrifuge balanced blood collection tubes for 10 min at 1500 to 2000 × g at 4 °C.
- Use the disposable transfer pipette to transfer the plasma (top layer) to a 15 mL conical tube (or 50 mL conical tube if collecting 30 to 100 mL of blood). Be careful not to disturb the buffy coat layer (the whitish layer in the middle) and the red blood cell layer (the red layer at the bottom).
- If more than one tube is collected, combine the plasma samples from all blood collection tubes into the same conical tube.
- Gently invert the conical tube 8–10 times to mix. Aliquot 250 μl to 1 ml into labeled microtubes or cryovials with O-ring-sealed screw leads. Residual aliquots can be saved and pooled as QC samples for repeated analysis.
- Store all aliquots upright in a specimen box in an -80 °C or colder freezer.

### Item 8: general procedures for buffy coat collection

Buffy coat contains most of the white blood cells and platelets of the anti-coagulated blood and is useful for a variety of clinical applications including genomic/genetic analysis. The remaining blood fractions after plasma collection can be used to collect buffy coat. To ensure high quality DNA, we recommend further enriching the buffy coats using a hypotonic solution to lyse the residual red blood cells. Figure 3C illustrates the general procedures for buffy coat collection.

- **Note: Prepare the following reagents ahead of time and store them at 4 °C.**
  - Ammonium chloride solution: 7.72 g/L
  - Ammonium bicarbonate solution: 0.79 g/L
  - Freezing mixture: TriPotassium Citrate: 17.8 g, Sodium Phosphate, monobasic: 2.4 g, Sodium Phosphate, dibasic: 2.8 g, Glycerin (Glycerol): 400 ml; bring volume to 1 L with distilled water.
- Freshly prepare **RBC lysis buffer** by combining 45 ml ammonium chloride solution and 5 ml ammonium bicarbonate solution.
- After removing the plasma (top layer) from the EDTA or heparin tubes, use another transfer pipette to draw the buffy coat (the whitish layer on top of the RBC layer) and place into the RBC lysis buffer tube (50 ml).
- Mix by pipetting up and down to separate any leftover cells from within transfer pipette.
- Cap the 50 ml tubes with lysis buffer + buffy coat and gently invert several times to mix.
- Incubate at room temp for **at least 20 min.**
- Add 10% bleach or Cavicide to the used blood tubes (lower layer with RBC) with leftover blood in them; discard in an appropriate biohazard bag.
- After 20 min incubation, centrifuge 50 ml tubes **at 4 °C for 20 min at 2500 rpm**
- After centrifuging, a white pellet will be visible at the bottom of the tube.
  - If no pellet is visible, centrifuge for an additional 20 min.
  - If pellet is visible, pour the red supernatant into a beaker filled with 10% bleach or Cavicide.
- Let pellet dry (approximately 10–20 min).
- Add 1 ml of freezing mixture to pellet. [**Freezing Mixture:** TriPotassium Citrate: 17.8 g, Sodium Phosphate, monobasic: 2.4 g, Sodium Phosphate, dibasic: 2.8 g, Glycerin (Glycerol): 400 ml; bring volume to 1 L with distilled water].
- Gently mix to break the pellet into single cell suspension.
- Transfer whole (cells + freezing mix) into cryotubes.
- Store at -80 °C for subsequent DNA isolation for genetic studies.

### Item 9: detailed step-by-step SOP for blood collection and processing

We have combined the points discussed above and our own experiences to develop an SOP for the handling of blood for biomarker measurements (see Additional file 1). This SOP, which was primarily developed to streamline handling procedures for neurodegenerative disease cohort studies at the University of Pittsburgh Alzheimer’s Disease Research Center and ancillary centers and studies, can also be used by other investigators including those who are new to blood biomarker studies. Importantly, the SOP is adapted to allow for the concurrent processing of whole blood into plasma/serum for protein biomarker evaluation, and buffy coat for genetic/genomic studies. Moreover, the design of the SOP enables multiple tube aliquots to be collected and stored, and residual volumes pooled for quality control (QC) purposes.

## Considerations for biobanking

### Item 1: storage tubes and temperature

To minimize analyte loss caused by adherence to the tube surfaces, it is recommended to use low protein binding microcentrifuge tubes or cryovials to store serum or plasma samples. For long-term storage, tubes with O-ring-sealed screws should be used to prevent evaporation. To prevent degradation, samples should be stored at ultra-low temperature freezers (-80 °C) or liquid N_2_ tanks instead of -20 °C freezers. If feasible, aliquots of the same sample should be stored in separate freezers to prevent complete loss in the case of a freezer failure.

### Item 2: freeze/thaw cycles

Several studies have investigated the impact of freeze/thaw cycles on blood biomarkers [107, 116, 141–143]. The overall results suggest that plasma AD biomarkers are stable for at least three freeze/thaw cycles. However, serum AD biomarkers may be more sensitive to freeze/thaw cycles. For example, serum Aβ40 level significantly decreased after any freeze/thaw cycle, while serum Aβ42 level showed a significant decrease at the third freeze-thaw cycle [107]. Therefore, to minimize the impact of freeze/thaw cycles, we recommend aliquoting samples in adequate volume, limiting the total number of freeze/thaw cycles to as few as possible, but never more than three.

### Item 3: transportation

To transport samples to different facilities, it is recommended to use abundant dry ice sufficient to last at least 24 h post the expected delivery time to avoid sample thawing due to delivery delays. If possible, use a courier that replenishes dry ice mid-way, and avoid weekend delivery. It is worth noting that dry ice inside storage tubes may change the pH of the specimen during thawing and potentially influence the assays [144]. Therefore, samples may be stored free of dry ice in -80 °C freezers for at least 24 h before thawing for measurement. To provide guidance to readers, we have compiled a list of recommended practices during blood transportation, created based on issues regularly encountered at a major biomarker laboratory.

- Include an accurate sample list (including accurate volumes). Ideally, provide a Microsoft Excel sheet (or similar) with columns for sample IDs, their corresponding volumes, and their location in the box. Adding a pictorial illustration of sample arrangements in the boxes is also helpful.
- Inform the receiving laboratory ahead of time so that they will keep a lookout and be able to receive and store them in good time. The receiving lab may need to find freezer space before your samples arrive. Therefore, it is important that they are informed ahead of time.
- Ensure that the package contains an adequate amount of dry ice. For long-distance transportation that spans multiple days, choose a reliable company that can refill the dry ice midway.
- Print labels using a computer, rather than handwriting them, to ensure better legibility.
- Use tubes with caps that do not become loose accidentally.
- Sort the samples in the order you want them analyzed. If you are unsure of the order, there are two main rules:
  1. If your samples are in groups: randomize them, so that all groups are represented in all analytical runs.
  2. If you have longitudinal samples, keep all samples from the same participant together and in the order in which they were collected. Ensure your sample coding reflects this ordering.
- Do the final sorting of the samples BEFORE you send them. It might take longer and be too time-consuming for the receiving lab to do it.

## Considerations during biomarker measurements

The quality of laboratory and clinical assays has greatly improved due to advances in instrument technology, particularly the use of automated equipment, which has led to higher reproducibility through standardized procedures. However, analytical errors/variations from various sources may still occur and render results unusable or confound study findings. Common sources of analytical errors/variations include instrument malfunction, operators’ failure to follow procedures, inherent batch-to-batch variation of the assay, and matrix interference. To minimize errors/variations, it is important to use good process controls and follow good laboratory practices. Below, we outline several considerations that are critical for ensuring high-quality results and monitoring/maintaining the long-term stability of the AD blood biomarker measurements.

### Item 1: preparation of samples for measurement

All blood specimens should be considered potential biohazards and handled with appropriate PPE. To ensure accurate measurement, it is crucial to homogenize samples before measurement. Samples should be completely thawed and mixed thoroughly. To minimize the impact of particulates that could clot cartridges or sample probes in the instrumentation, samples should be centrifuged before dispensing into a measurement container. It is also important to minimize the bubbles during pipetting. Any remaining samples should be promptly returned to the -80 °C freezer to minimize protein degradation. Place a dot on the tube lid with a permanent marker after each freeze-thaw cycle and keep track of the total number of cycles in updated inventory of samples. It is also recommended to keep samples with the same number of freeze-thaw cycles together and separate from the original, un-thawed samples.

### Item 2: inclusion of calibrators

Despite significant technological advances, batch-to-batch variation remains an inherent issue in clinical chemistry. Contributing factors may include variations in instrument performance, changes in reagents and consumables, operator variability, etc. To address this issue, calibrators should be included in every batch to help correct for batch effects. Some platforms, such as Lumipulse, have been built to circumvent this issue such that there is an internal, manufacturer-provided calibration curve against which all sample results are plotted. However, this approach only works if the manufacturer can adjust all reagent batches to perform equivalently to the initial batch used to generate the built-in curve. The acceptance criteria for the calibration curve must be predefined to ensure accuracy and consistency. Although these criteria may vary depending on the platform used, certain parameters such as the regression coefficient, the variance between predicted and actual concentrations, and the repeatability for each calibrator should be considered.

### Item 3: inclusion of QC samples

**Fig. 4:**
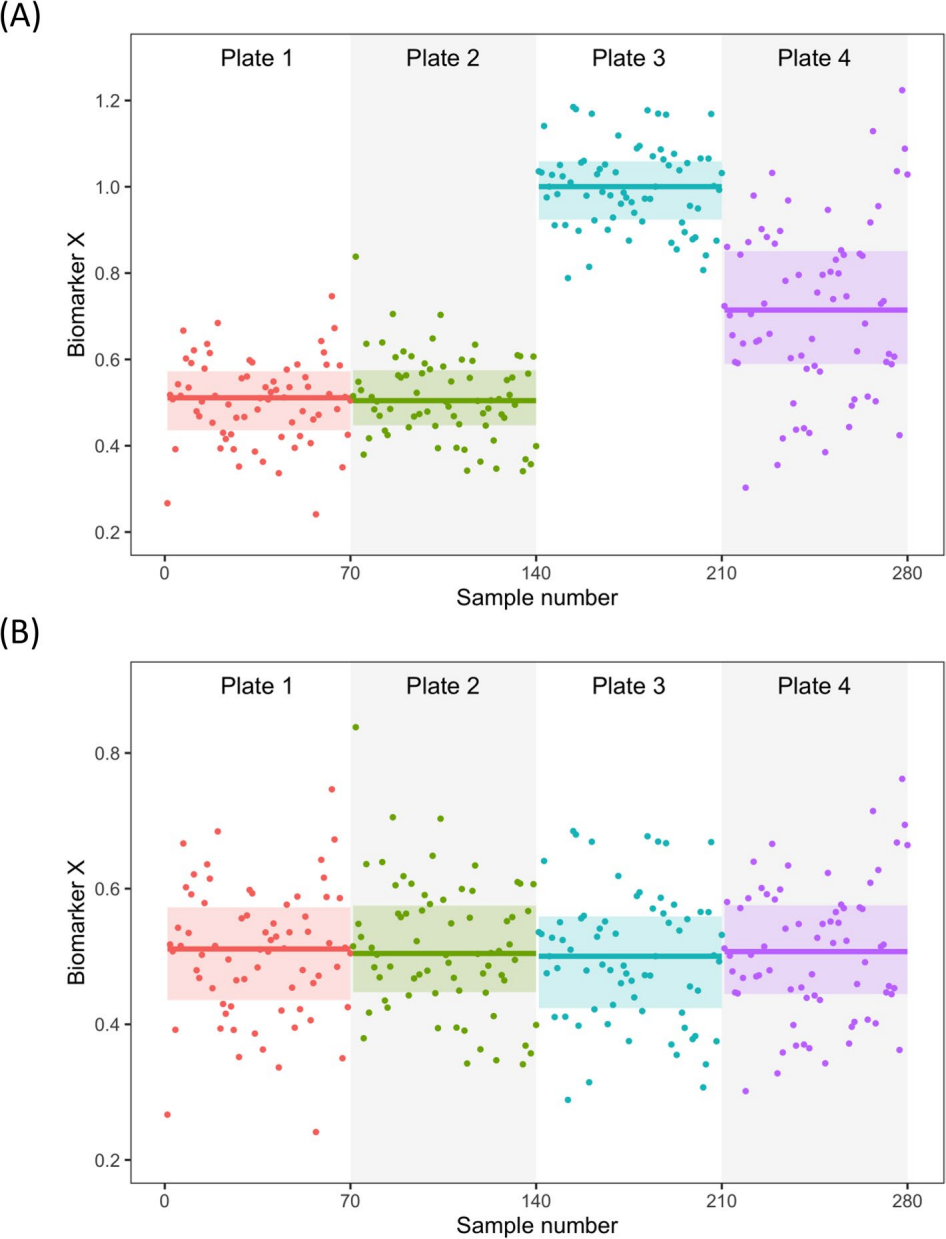
Visualization of data generated from measuring a hypothetical biomarker on 280 samples spread across four plates. The figure shows the results before (**A**) and after (**B**) adjustment based on the signals generated for identical aliquots of the same quality control samples measured on each plate, assuming all other key variables remain the same

QC samples play a critical role in process control and should be included in every analytical run to evaluate assay performance and address potential errors. At a minimum, QC samples should be run in duplicates at the beginning and end of each analytical run. These samples should be selected in a way that they cover a range of concentrations across the standard curve or the typical range of concentrations for the measured sample values. It is typically advisable to use use three QC samples – low, average, and high concentrations relative to the assay standard curve. For optimal results, the same lot of QC samples should be used over time to help detect system or operator performance changes. Whenever possible, QC samples should be the same specimen types as the test samples. When switching between batches of QC samples, bridging is recommended – the relative comparison of QC samples to adjust the biomarker distribution on one plate to the other. These factors can then be applied to all samples measured on each plate/run to normalize the entire dataset. In Fig. 4, we present a hypothetical example of a cohort consisting of 280 samples that were analyzed across four plates for Biomarker X. As shown in Fig. 4A, the mean and overall data distribution of the uncorrected results from plates 1 and 2 appear similar, while plates 3 and 4 show a noticeable shift (simulation datasets in Additional file 2). However, after normalization using the plate-specific scaling factors based on QC readings, as shown in Fig. 4B, the results from all four plates become more comparable.

Similar to calibrators, a predefined set of criteria should be applied to determine whether a run passes or fails. Key parameters to consider include the deviation from target values established through prior repeated analyses and the reproducibility of the QC measurements. An article by Schindler et al. [145] which tracked CSF assay performances over approximately a decade sheds light on this issue and potential corrective factors.

### Item 4: sample measurement order

Batch-to-batch variation and within-batch run order effects can bias results, leading to erroneous conclusions. One way to address these issues is to randomize the order of sample analysis. There are various methods of randomization, including simple randomization, block randomization, stratified randomization, and covariate adaptive randomization [146]. Simple randomization involves using a single random sequence and is easy to implement, for example, by using the random number generator in Excel. However, simple randomization may result in an unequal distribution of groups and may not be suitable for studies with a small sample size (*n* < 200). Block randomization addresses this issue by randomly assigning an equal number of samples from each group to each block and is more appropriate for small-size studies. For studies with multiple covariates, stratified randomization or covariate adaptive randomization may be necessary to control the potential uneven distribution of covariates. Additionally, for longitudinal measures, we recommend measuring all time points for the same participant simultaneously to minimize the impact of batch variation.

### Item 5: sample blinding

Knowledge of grouping information may bias the analysis results and taint the data. Whenever possible, scientists performing the measurements and the analysts/statisticians who work with the ensuing data should be blinded from grouping allocation until measurement results have been finalized. A third party should perform the randomization to generate sample analysis orders.

### Item 6: assay order

If multiple biomarkers are to be measured from the same aliquots, it is best practice to start with the biomarker most sensitive to degradation/denaturation from repeated freeze/thaw cycles. For example, among the core AD blood biomarkers, Aβ40 and Aβ42 tend to be most prone to degradation and should therefore be measured first, if possible, followed by t-tau, p-tau, BD-tau and finally GFAP and NfL. In addition, using multiplexed assays, if available, can help minimize the number of freeze/thaw cycles required.

### Item 7: the use of bridging samples

For discovery research, it is recommended to process all samples at the same time with the same batch of reagents. However, in cases where it is unavoidable to change the reagent lots or run the analysis at different times or in various laboratories, harmonization can be achieved by repeating the measurement for bridging samples. Ideally, bridging samples should be from the same sample cohort and cover the full range of values of the study samples. The bridging samples are analyzed using the same reagents and consumables for each batch, allowing the determination of batch-specific normalization factors. The same approach described in Fig. 4 for normalizing run data from different plates is applicable here to between-batch normalization.

### Item 8: longitudinal samples

Longitudinal studies, which involve continuous tracking of the same individuals over time, are commonly employed to assess the influence of various interventions or risk factors on disease outcomes. In contrast to cross-sectional studies, which capture snapshots of participants at a single point in time, longitudinal studies typically require an extended duration to collect specimens for the entire study. In some cases, researchers may be able to wait until all samples are collected to run them simultaneously. However, there are instances where running batches of samples along the way becomes necessary to gain preliminary insights into the data. To minimize the impact of temporal variation, proper control measures must be implemented to monitor and maintain the long-term stability of measurements. Several practical guidelines, proposed by Palmqvist et al. for AD CSF biomarkers, can be adopted for blood AD biomarkers [78]. These include controlling preanalytical variables, following good laboratory practices, utilizing calibrators and QC samples, and assessing the batch effect through batch-bridging. Ideally, one would like to use the same QCs throughout a longitudinal study. However, if this is not possible, it is important to perform batch-bridging and re-run samples from the previous analysis round to normalize runs. Therefore, it is crucial to properly plan for the longitudinal studies, including stocking up reagents (including QC and bridging samples) and consumables for the entire study if shelf life is allowed. While most blood AD biomarker research has been conducted in research settings, there is an increasing number of studies being initiated to evaluate the use of these biomarkers in real clinical practice, where specimens are continuously analyzed. Maintaining measurement stability becomes even more important in these real-world settings.

## Considerations for result reporting

### Item 1: demographic and clinical information

To minimize potential bias caused by demographic and clinical factors, it is important to include the distribution of available demographic and clinical characteristics among groups in the report, along with their associated statistical findings. These information help identify potential moderating variables and adjust for their effect in the analysis. Additionally, including this information in the report allows better comparison with results from different studies.

### Item 2: full description of methods

To facilitate the coherent pooling of data in future meta-analyses, it is important to include detailed descriptions of blood collection/handling protocols and laboratory assay protocols in the Method section of peer-reviewed publications. In addition, as preanalytical variables, such as blood draw devices and parameters during blood processing, may interfere with AD biomarker measurements, it is important to describe these variables and to indicated if they were changed during the study.

### Item 3: disclosure of assay performance

It is important to include assay performance in the publication to enable other researchers to evaluate the quality of the reported results. Multiple attributes can be useful for assessing assay performance, such as calibration curves’ *R*-value, linearity range, the limit of detection, and limit of quantification, as well as QC performance measures such as repeatability and intermediate precision. Moreover, the reports should also disclose if plate adjustments, such as with the use of bridging samples or QC samples, have been performed to account for batch variation.

## Unresolved issues

### Certified reference materials

A major source of confusion in the field is that blood biomarker assays for the same analyte (e.g., plasma p-tau181 or p-tau217) from various sources return very different numerical values in terms of concentrations. We have sought to explain the analytical reasons behind this phenomenon – that it is often due to the use of different calibrators as well as variability of signals from the technical platforms [13]. However, the issue limits the comparability of assay results across platforms, which might become even more acute once cutpoints are generated. The use of certified reference materials could provide a solution, by providing a focal point for comparing and calibrating the standards used in the different assays. An example of the use of certified reference materials in mitigating between-assay bias was demonstrated by the application by Boulo S et al. in CSF samples [147].

### Real world applications

Despite excellent performances of blood biomarkers demonstrated in many publications, we should be mindful that the results have mostly come from highly selected research cohorts that additionally have prior confirmation of pathology using CSF or neuroimaging biomarkers or even sometimes both. As we venture into applying blood biomarkers as potential first line screeners in clinical practice and therapeutic trials, it is important to note that this might be the real “test drive” for the biomarkers. As such, their performances may not be as stellar as observed for multiple reasons. This includes the much lower prevalence of AD pathophysiology in the community compared with the research cohorts, and the high heterogeneity of diseases presented in the clinics contrary to those included in defined cohorts. Moreover, such heterogenous cases would not benefit from prior categorization using CSF and neuroimaging biomarkers as often done for the existing cohorts that have recorded high accuracies.

### Cutpoints

Clinical use of blood biomarkers is much anticipated. However, it is often forgotten that one of the fundamental factors that need addressing prior to this happening is the generation and validation of assay thresholds. Such threshold points would not only be based on the detection of amyloid and/or tau pathology but would additionally investigate potential clinical scenarios such as if and how the set cutpoints respond to given demographic and clinical variables.

## Conclusion

The blood AD biomarker research field has experienced rapid growth in recent years, with numerous studies highlighting the potential of blood-based AD biomarkers in supporting clinical decision-making and accelerating therapeutic development. We have outlined in this review general guidelines for the various steps involved in blood AD biomarker measurement, to promote good laboratory practices to minimize analytical errors and facilitate the development of standardized protocols that can improve reproducibility and enable cross-validation across different research centers. Table 2 provides a summary of the important preanalytical, analytical and post-analytical factors to consider in AD blood biomarker research. However, further research is needed to fully understand the impact of preanalytical and analytical variables, and these guidelines should be updated as new research findings become available. Finally, we provide an adaptable SOP that can be applied to blood collection, processing, and downstream handling in biomarker evaluations.

**Table 2:** List of important factors to consider in AD blood biomarker research

**Pre-analytical**	**Analytical**	**Post-analytical**
• Participant preparation: fasting status, blood draw time • Plasma vs. serum • Blood draw devices • Blood collection tubes • Blood draw order • Blood tube filling height • Blood tube inversion • Serum clotting time • Time from blood draw to centrifugation • Centrifugation parameters, including speed, time, and temperature • Time from centrifugation to storage • Storage temperature • Microtubes for specimen storage • Freeze/thaw cycles • Transportation temperature • Proper tube labeling to minimize error	• Sample thawing and homogenization • Centrifugation for particulate removal • Prompt storage of unused specimen • Inclusion of calibrators • Inclusion of QC samples • Sample measurement order • Sample blinding • Assay orders for multi-assay studies • Inclusion of bridging samples • Order for longitudinal samples • Batches/lots for reagent and consumable • Selection of analytical platforms	• Evaluation of calibrator curves • Evaluation of QC results • Adjustment of potential moderating variables • Full description of methods • Disclosure of assay performance

### Supplementary Information

**Additional file 1.** Step-by-step blood specimen collection procedures.

**Additional file 2.** Simulation datasets used to generate the plots in Fig. 4.

## Data Availability

All the data are included in the manuscript and the supporting information.

## Abbreviations

Aβ: Amyloid-beta
AD: Alzheimer’s disease
BD-tau: Brain-derived tau
CSF: Cerebrospinal fluid
DBS: Dried blood spot
DPS: Dried plasma spot
ECL: Electrochemiluminescence
EDTA: Ethylenediaminetetraacetic acid
ELISA: Enzyme-linked immunosorbent assay
GDPR: General Data Protection Regulation
GFAP: Glial fibrillary acidic protein
GLP: Good laboratory practice
HIPAA: Health Insurance Portability and Accountability Act of 1996
IME: Interdigitated microelectrode sensor
IMR: Immunomagnetic reduction
IP-MS: Immunoprecipitation coupled with mass spectrometry
IV: Intravenous
MRI: Magnetic resonance imaging
MS: Mass spectrometry
MSD: Meso Scale Discovery
NfL: Neurofilament light-chain
p-tau: Phosphorylated tau
PET: Positron emission tomography
PPE: Personal protective equipment
QC: Quality control
Simoa: Single-molecule array
SOP: Standardized operating procedure
SST: Serum separator tubes
t-tau: Total tau
