# Supplementary material for: Alzheimer blood biomarkers: practical guidelines for study design, sample collection, processing, biobanking, measurement and result reporting
Source: Mol Neurodegener. 2024 May 15;19:40. doi: 10.1186/s13024-024-00711-1 (PMC11095038; doi:10.1186/s13024-024-00711-1)
Supplement: Supplementary file 1 — Additional file 1. Step-by-step blood specimen collection procedures. [file 13024_2024_711_MOESM1_ESM.docx]

**Additional file 1**

**UnIVERSITY OF Pittsburgh**

**Alzheimer’s Disease Research Center (ADRC)**

**Standard Operating Procedure (SOP)**

**For Blood Specimen Collection and Processing**

1. **Purpose**

Proper blood specimen collection and handling process is critical to ensure the quality of laboratory test results. This SOP describes the procedures for collecting, aliquoting, and storing blood specimens for ADRC studies. These procedures should be followed by all study personnel responsible for processing blood specimens for the ADRC.

1. **Blood specimen collection chart**

| Collection tube | Number of tubes | Blood volume | Specimen type | Aliquot volume | Number of aliquots |
| --- | --- | --- | --- | --- | --- |
| EDTA (lavender-top) blood collection tubes (10 ml) | Up to 3 | 10 ml each | plasma | 1 ml | Up to 10 |
|  |  |  | Buffy coat |  | Up to 2 |
| Plain red top (4 ml; plastic) | Up to 2 | 4 ml each | Serum | 1 ml | Up to 3 full aliquots and one residual aliquot |
| PAXgene® Blood RNA tube | 1 | 2.5 ml | RNA | 2.5 ml | 1 |

Note: The PAXgene Blood RNA tube contains 6.9 ml of additive that stabilizes the intracellular RNA molecules and should be drawn last in the phlebotomy procedure.

1. **General precautions**

- Gloves must be worn for all sample handling, including removing the rubber stopper from the blood tubes, centrifugation, pipetting, disposal of contaminated tubes, and cleanup of spills.
- All waste must be properly disposed of in biohazard containers. For the used blood tubes, add 10% bleach or Cavicide to the leftover blood in them before discarding them in the biohazard bag.
- Biohazard labels should be affixed to all equipment used for blood specimen collection.
- Patients/participants should be identified with unique non-descriptive identification numbers to prevent the breach of private information.
- Precautions need to be taken to reduce the risk of hemolysis. Using needles with inappropriate gauges, puncture site not completely dry from disinfectant, excessive pulling during the blood draw, excess anticoagulant to blood ratio due to underfilling, or mixing the blood collection tubes too vigorously after the blood draw are some factors that may increase the risk of hemolysis.

**4. Required equipment and materials**

- Centrifuge with swinging bucket rotor (capable of ≥ 2000 x g with refrigeration to 4°C)
- -80°C freezers for storage
- Single channel mechanical pipette, 100–1000 µl adjustable volume
- Pipette tips (1000 µl)
- Disposable sharps containers with lid
- Biohazard waste container
- Bleach or similar disinfectant
- Tube rack for blood collection tubes
- Styrofoam box for transport
- Ice packs for transport in a hot environment
- Disposable transfer pipets (7.7 mL; Fisher Scientific, Cat # 13-711-7M)
- EDTA (lavender-top) blood collection tubes (10 mL; BD Biosciences Cat# 366643): up to 3 per participant
- PAXgene® Blood RNA Tube (2.5 ml; BD Biosciences Cat # 762165): one per participant
- Plain red top serum tubes (4 ml; BD Biosciences Cat # 367812): up to 2 per participant
- 15 mL polypropylene conical tubes: need one for serum and one for plasma
- 50 ml polypropylene conical tubes: one for one or two 10 ml EDTA tubes; two for three 10 ml EDTA tubes
- Cryovials
- Cryovial tube labels

**5. Required reagents**

- Ammonium chloride solution: 7.72 g/L
- Ammonium bicarbonate solution: 0.79 gm/L
- Freezing mixture
  - Tripotassium citrate: 17.8 g
  - Sodium phosphate monobasic: 2.4 g
  - Sodium phosphate dibasic: 2.8 g
  - Glycerin (Glycerol): 400 ml
  - Bring volume to 1 L with distilled water.
- **Note: the reagents should be prepared ahead of time and stored at 4^o^C.**

**6. Preparation the day before the blood draw**

- **Gather patient information:** Obtain the participant ID number, blood draw date, and time from the clinical core staff before the blood draw. **Note: Clinical core staff must record the study name, the participant ID number, the blood draw date, and the time on the EDTA blood collection tubes.**
- **Prepare labels:** Use a label printer to print the labels according to the format below.

**
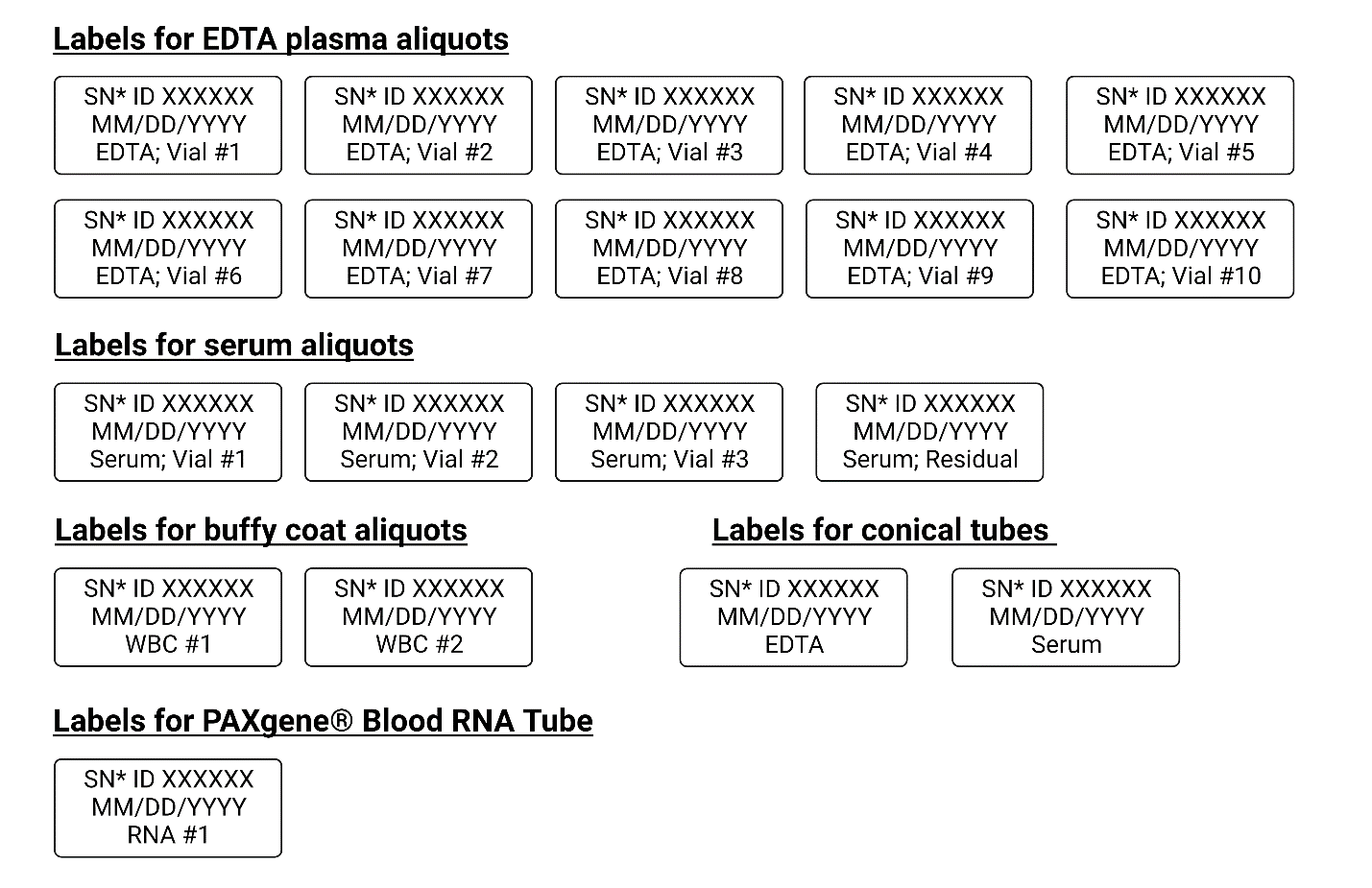
**

**Note:** SN* refers to the abbreviation of the study name.

- **Check supplies:** Check to make sure there are enough supplies.

1. **Blood Collection procedures in the clinics**

- **Step 1:** Use a permanent marker to label all blood collection tubes with the study name, participant’s ID number, blood draw date, and time.
- **Step 2:** Draw blood according to the best practices in phlebotomy. When possible, use straight needle venipuncture with 21-gauge needles. In case alternative phlebotomy procedures are employed, document the variations in the Specimen Processing Information Form. **Critical: Allow the tubes to be filled until the vacuum is exhausted and blood flow ceases to ensure a proper additive-to-blood ratio.**
- **Step 3:** Immediately after blood collection, gently mix the blood by inverting the tubes (180-degree turn) according to the table below. **Critical: Mixing the blood collection tubes too vigorously may increase the risk of hemolysis.**

| Collection tube | Number of inversions |
| --- | --- |
| EDTA (lavender-top) blood collection tubes | 8 to 10 times |
|  |  |
| Plain red top (4 ml; plastic) | 5 times |
| PAXgene® Blood RNA Tube | 8 to 10 times |

- **Step 4:** Place the blood collection tubes upright on a tube rack and then place the tube rack in a Styrofoam box for transferring to the processing lab. **Critical: Place ice packs in the Styrofoam box to ensure a cool environment if blood tubes are transported in a hot environment. Avoid direct contact of blood collection tubes with the ice, which may cause the rupture of blood cells and increase the risk of hemolysis.**

**
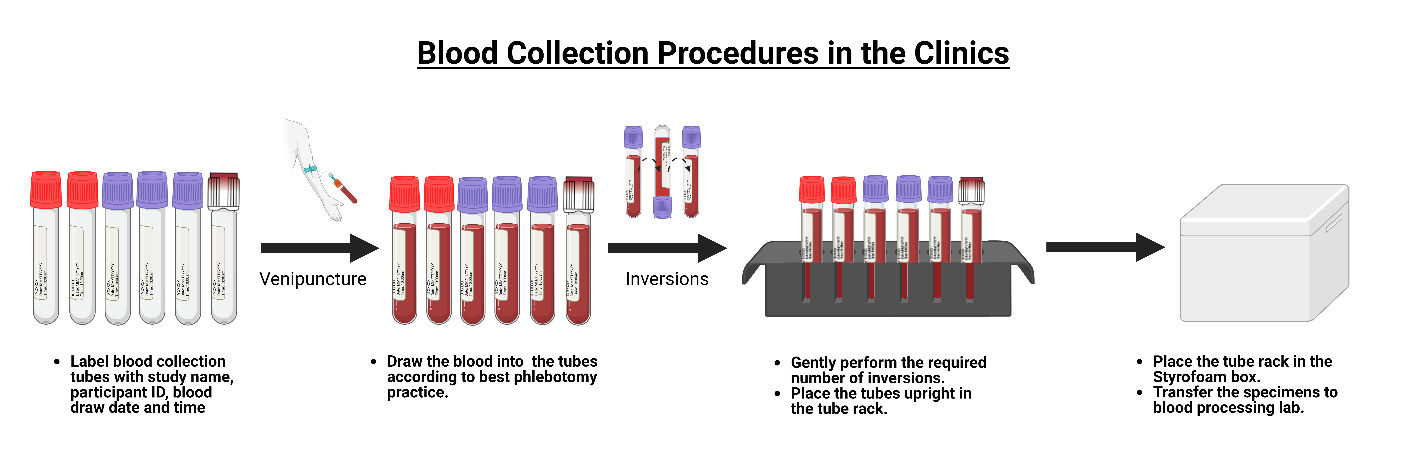
**

1. **Blood processing procedures in the wet lab**
2. **Processing plasma from EDTA tubes**

- **Step 1:** Centrifuge appropriately balanced EDTA tubes for 10 minutes at 2000 x g and 4°C. **Critical: EDTA blood samples should be centrifuged within one hour of blood collection. For optimum reproducibility, aim to start centrifugation at exactly 1 hour from sample collection; document the exact blood draw to the centrifugation time interval for every sample in the Specimen Processing Information Form (Appendix A). Centrifugation must be performed using a swing bucket rotor instead of a fixed-angle rotor to ensure stable plasma separation.**
- **Step 2:** After centrifugation, use the disposable transfer pipette to transfer the plasma (top layer) from blood collection tubes to a 15 mL conical tube. Leave about 5-10% behind to ensure no cross-layer contamination. Save the blood tubes in the refrigerator for buffy coat collection. Affix the pre-printed label to the conical tube. **Record the hemolysis scale in the Specimen Processing Information Form according to the US Center for Disease Control and Prevention’s Hemolysis Reference Palette (Appendix B).**
- **Step 3:** Invert the 15 mL conical tube 8 to 10 times. Aliquot 1 mL plasma into each cryovial for up to total 10 aliquots. Firmly affix the cryovial labels to all cryovials.
- **Step 4A:** Place the labeled cryovials in the freezer boxes and store them in -80^o^C freezers. **Note: if feasible, split the cryovials into two freezer boxes and store them in separate -80°C freezers to avoid catastrophic loss due to freezer breakdown.**
- **Step 4B:** Place the conical tube in the quality control (QC) rack in the -80°C freezer. The residual plasma in the conical tube will be pooled and used as QC samples to evaluate signal reproducibility during blood biomarker measurements.
- **Step 4C:** Document processing parameters in the Specimen Processing Information Form and update the "Specimen Inventory Log" and “Freezer Log”.


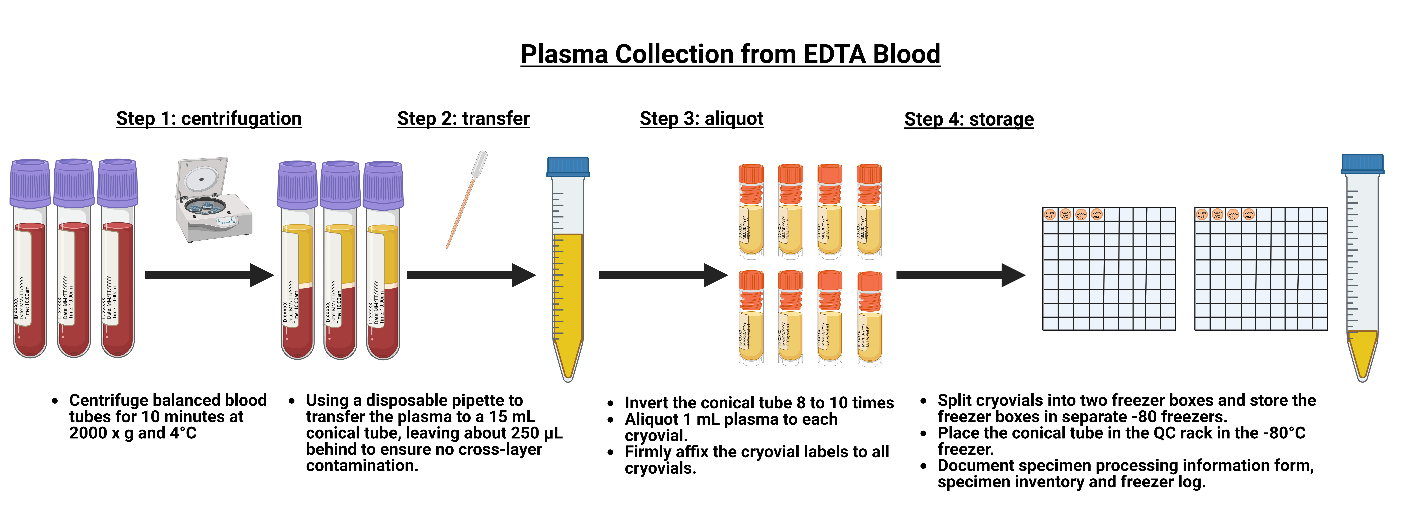


1. **Processing buffy coat from EDTA tubes**

- **Step 1:** Arrange an appropriate number of 50 ml conical tubes. Fill each with 50 ml red blood cell (RBC) lysis buffer (45 ml ammonium chloride + 5 ml ammonium bicarbonate).
- **Step 2:** Retrieve the EDTA tubes from the refrigerator saved during plasma processing. Carefully remove and discard the residual plasma in the top part of the blood collection tubes with the disposable pipette. With another transfer pipette, draw the buffy coat (white layer on the top of the RBC layer) and place it into the RBC lysis buffer tube (50 ml). Mix by pipetting up and down to separate any leftover cells from within the transfer pipette. **Note: up to 2 EDTA tubes FROM THE SAME PARTICIPANT AND THE SAME BLOOD DRAW per RBS lysis buffer tube.**
- **Step 3:** Cap the 50 ml conical tubes with RBC lysis buffer+WBC and gently invert several times to mix. Incubate the tubes at room temp for at least 20 minutes.
- **Step 4:** After the incubation, centrifuge the 50 ml conical tubes at 4°C for 20 minutes at 2500 rpm. A white pellet will be visible at the bottom of the tube. If no pellet is visible, centrifuge for an additional 20 minutes.
- **Step 5:** Pour the red supernatant into a beaker filled with disinfectant. Let the pellet dry (approximately 10 – 20 minutes).
- **Step 6:** Add 1 ml of freezing mix to each pellet. Use a mechanical pipette to gently mix to break the pellet into single-cell suspension. Combine mixtures from both tubes if more than two 10 ml EDTA tubes are used (from the same participant and the same blood draw).
- **Step 7:** Aliquot 1 mL of the mixtures (cells+ freezing mix) into cryo tubes. Firmly affix the cryovial labels to all cryovials. Store all vials at -80°C freezer.


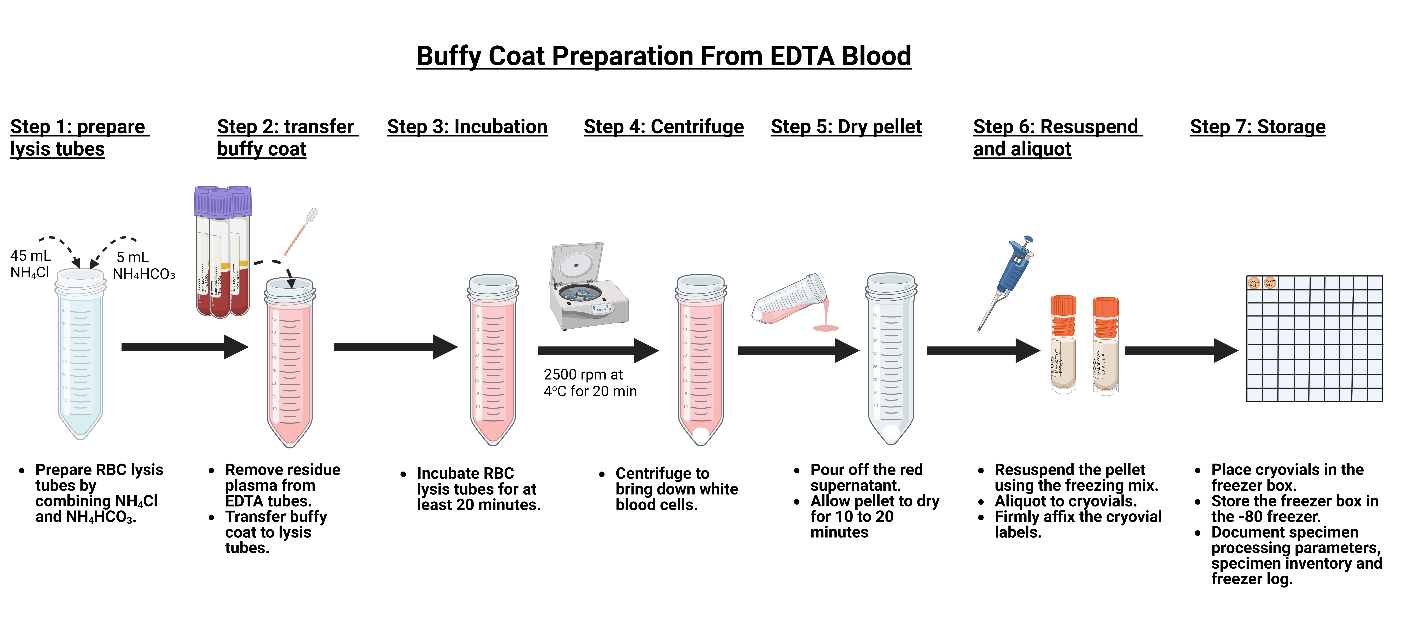


1. **Processing serum from red top tubes**

- **Step1:** Place the filled red top blood collection tubes upright at room temperature for 30 to 60 minutes to allow the clot to form. **CRITICAL: If the blood is not centrifuged immediately after the clotting time, the tubes should be refrigerated (4^o^C) for no longer than 2 hours.**
- **Step 2:** Centrifuge the balanced clotted tubes for 10 minutes at 2000 x g at 4^o^C.
- **Step 3:** Use the disposable transfer pipette to transfer the serum (top layer) from blood collection tubes to a 15 mL conical tube. Leave about 5-10% behind to ensure no cross-layer contamination. Affix the pre-printed label to the conical tube. **Record the hemolysis scale in the Specimen Processing Information Form according to the US Center for Disease Control and Prevention’s Hemolysis Reference Palette (Appendix B).**
- **Step 4:** Invert the 15 mL conical tube 8 to 10 times. Aliquot 1 mL serum into each cryovial (red lid) for up to a total of 3 full aliquots. Save the residual aliquot in a cryovial (orange lid). Firmly affix the corresponding cryovial labels to all cryovials.
- **Step 5:** Place the labeled cryovials in a freezer box and store them in a -80^o^C freezer. **Document processing parameters in the Specimen Processing Information Form (Appendix A) and update the "Specimen Inventory Log" and “Freezer Log”.**


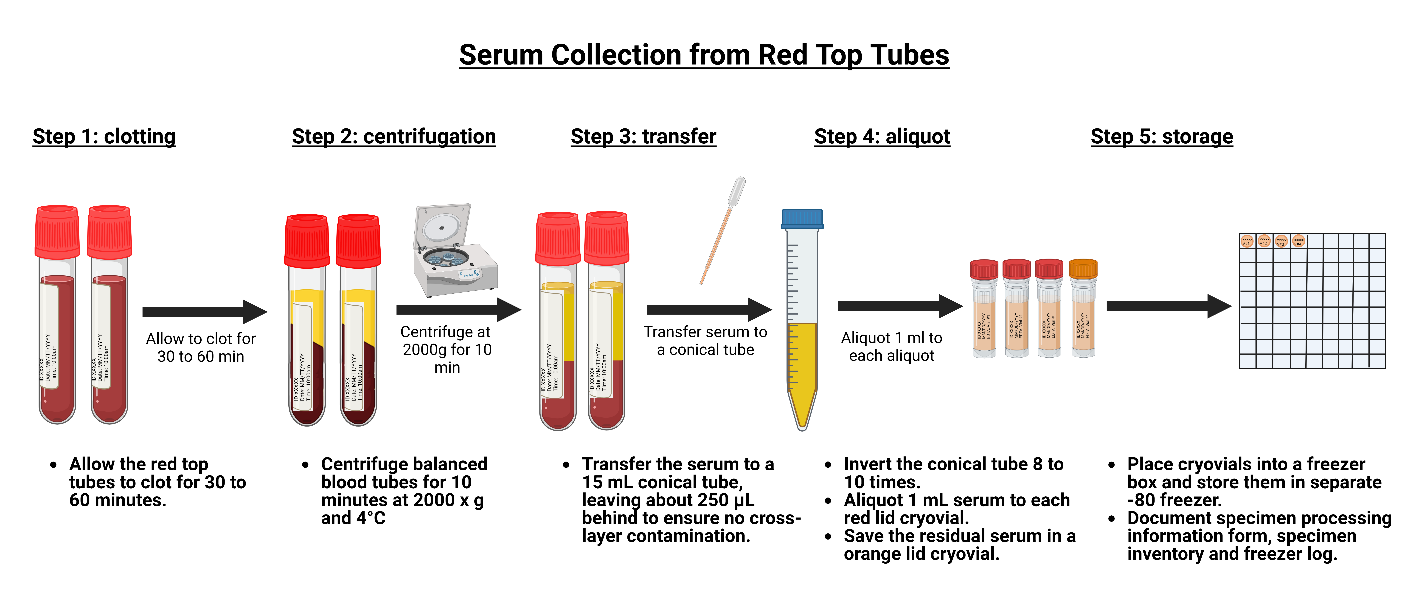


1. **Processing PAXgene® Blood RNA Tube**

- **Step 1:** After the blood draw and mixing by 8 to 10 inversions, keep the PAXgene Blood RNA Tube upright in a tube rack at room temperature for 24 hours. **Note: BD Biosciences recommends keeping the tubes at room temperature for 2 to 72 hours before storage.**
- **Step2:** Transfer the PAXgene Blood RNA Tube upright to a wire rack at -20^o^C freezer for storage. **Note: Do not use a Styrofoam™ tray for storage as this may crack the tubes.**

**
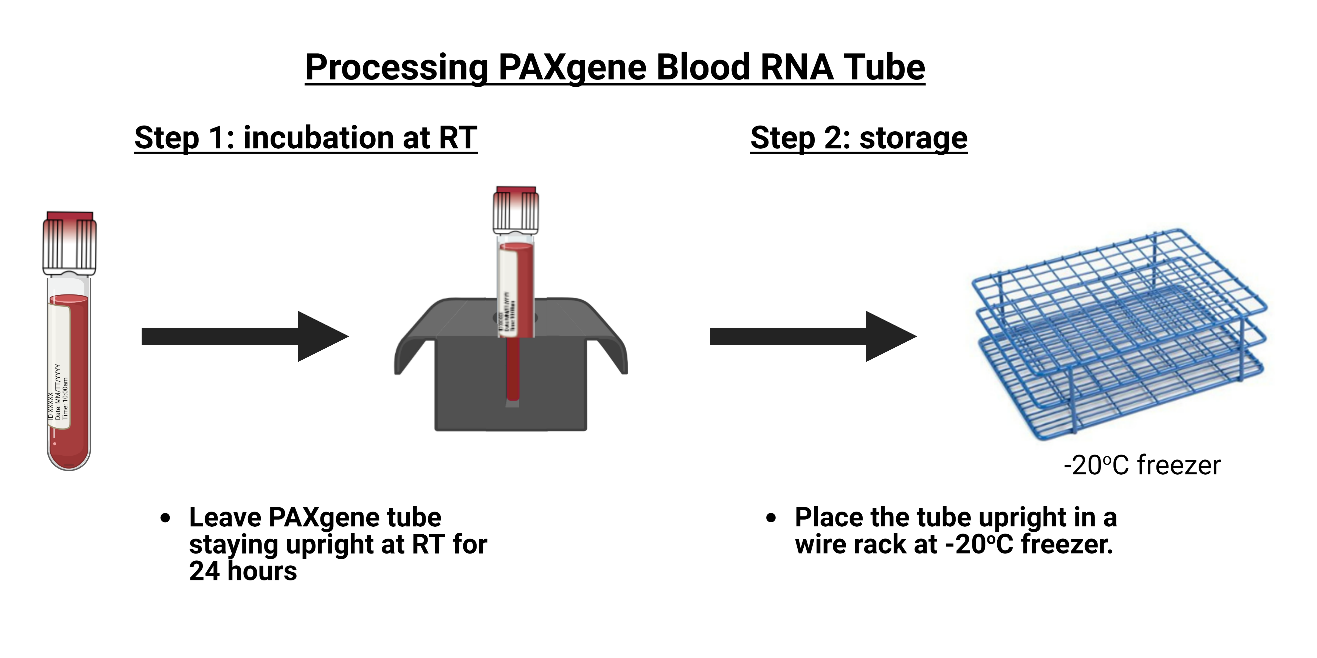
**

# Appendix A

**Specimen Processing Information Form**

Date: ____________ Participant ID: _______________

**Blood Collection:**

Time of Draw: _______________ Blood drawn by: _______________

**Plasma Processing** (Processed by: _______________)

| **Centrifugation parameters** | | | |
| --- | --- | --- | --- |
| Time started | Duration (min) | Temp (^o^C) | Force (x g) |
|  |  |  |  |
|  | | | |
| **Full aliquot volume (mL)** | **Number of full aliquots** | **Residual volume (mL)** | **Hemolysis scale** |
|  |  |  |  |

**Serum Processing** (Processed by: _______________)

| **Clotting** | |
| --- | --- |
| Start time | End time |
|  |  |

| **Centrifugation parameters** | | | |
| --- | --- | --- | --- |
| Time started | Duration (min) | Temp (^o^C) | Force (x g) |
|  |  |  |  |

| **Full aliquot volume (mL)** | **Number of full aliquots** | **Residual volume (mL)** | **Hemolysis scale** |
| --- | --- | --- | --- |
|  |  |  |  |

Notes:______________________________________________________________________________________

# Appendix B

**Hemolysis Reference Palette Guide**

[**https://www.cdc.gov/ncezid/dvbd/pdf/Hemolysis_Palette_Bookmark-P.pdf**](https://www.cdc.gov/ncezid/dvbd/pdf/Hemolysis_Palette_Bookmark-P.pdf)

**
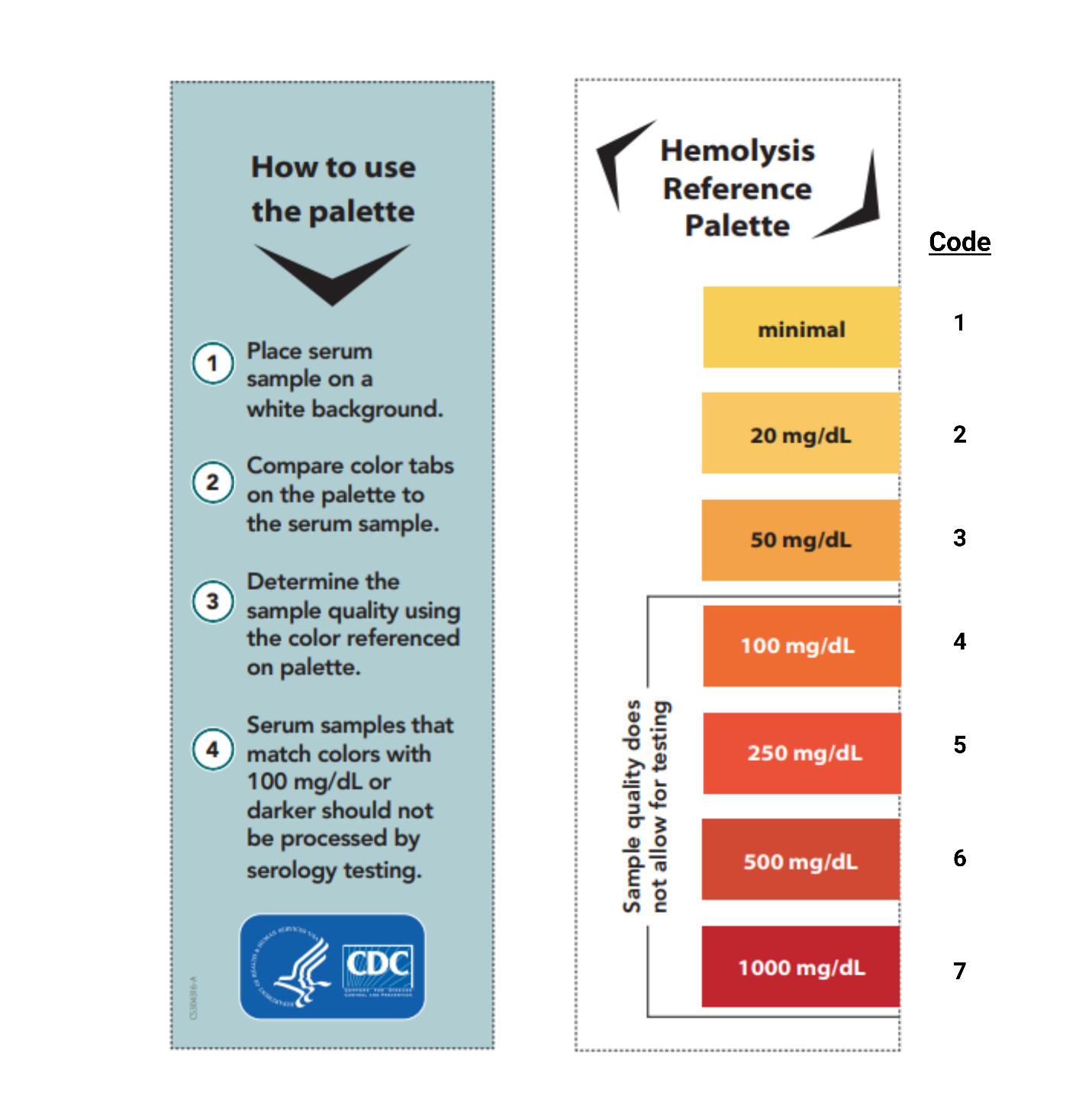
**
